# Prioritizing vulnerable populations and women on the frontlines: COVID-19 in humanitarian contexts

**DOI:** 10.1186/s12939-020-01186-4

**Published:** 2020-05-13

**Authors:** Vandana Sharma, Jennifer Scott, Jocelyn Kelly, Michael J. VanRooyen

**Affiliations:** 1 Harvard T.H. Chan School of Public Health, 677 Huntington Avenue, Boston, MA 02115 USA; 2 Harvard Humanitarian Initiative, Cambridge, MA USA; 3 Harvard Medical School, Boston, MA USA; 4 Beth Israel Deaconess Medical Center, Boston, USA; 5 Brigham and Women’s Hospital, Boston, USA

**Keywords:** COVID-19, Humanitarian contexts, Women, Gender, Essential workers, Displaced populations, Gender bias, Humanitarian organizations

## Abstract

The COVID-19 outbreak has been declared a global pandemic and cases are being reported among displaced populations that are particularly vulnerable to infection. Humanitarian workers on the frontlines of the response are working in some of the most challenging contexts and also face elevated risk of contracting COVID-19 and potential stigmatization or violence in the community. Women humanitarians may be at even greater risk, but their protection is dependent on organization-specific policies and procedures. Without gender balance in leadership positions, the specific needs of women may not be prioritized and women may not be included in decision-making or design of responses. Ensuring gender equitable access to personal protective equipment and information is imperative, but additional measures must be put into place to ensure the protection of women on the frontlines while reducing COVID-19 deaths and adverse health effects among displaced populations.

## Commentary

The coronavirus disease 2019 (COVID-19) outbreak was declared a pandemic on March 11, 2020 by the World Health Organization (WHO) and has spread to 185 countries [1]. The UN Refugee Agency (UNHCR) reports 96 of the affected countries are refugee-hosting countries [2], and COVID-19 cases are now being reported among displaced communities [3]. Displaced populations, including refugees, are particularly vulnerable to infection given crowded living conditions, limited access to healthcare, safe water and sanitation and other factors including poorer underlying health and nutritional status [4]. Humanitarian workers on the frontlines of this response are working in some of the most challenging contexts and also face elevated risk of contracting COVID-19.

Globally, governments are responding to the pandemic through measures such as restricting refugee movement, tightened border control and lockdown of displacement camps [3]. These measures may pose substantial challenges to the continued provision of humanitarian assistance and life-saving services. Humanitarian organizations have already reported reduced access to affected populations [5], and disruption of humanitarian operations [6]. The UN Secretary-General, António Guterres, has emphasized the need for a global, coordinated response to COVID-19 that addresses the specific needs of displaced populations and has urged countries to designate humanitarian professionals as ‘essential’ workers, given their role in providing indispensable services [7].

Essential workers face heightened risks of contracting COVID-19 through their high levels of exposure and inadequate access to personal protective equipment (PPE), as well as increased risks of violence in the workplace, and potential stigmatization or violence in the community [8]. In humanitarian contexts, there are reports of backlash against aid workers, stemming from fear and stigma related to COVID-19 [5]. Women on the frontlines may be at even greater risk. There are anecdotal reports of female health workers being expelled from their apartments in the Democratic Republic of the Congo and the Philippines due to fear they may be carrying COVID-19 [8]. Similar reports of harassment of female frontline health providers caring for patients have also arisen in India [9]. Gendered imbalances in access to resources may also result in male workers being prioritized for PPE [8]. In Italy and Spain, gender differences in COVID-19 infections among health workers are evident as twice as many female than male health workers have been infected with the virus [10]. While the COVID-19 Global Humanitarian Response Plan emphasizes the consideration of gender, the focus is on affected populations [6]. Protection of female humanitarian workers is dependent on organization-specific policies and not standardized across the sector.

However, many humanitarian organizations lack policies and procedures that address the distinct needs of female frontline workers. While women comprise more than the majority of humanitarian workforce, only 25% of leadership positions in humanitarian organizations are held by women [11]. Without gender balance in leadership positions, the specific needs of women may not be prioritized and women may not be included in decision-making or design of responses. This may lead to gender-neutral policies or even gender-discriminatory practices. Where gender-equitable policies do exist, lack of effective implementation or enforcement is common [8]. Gender biases, at both individual and organizational levels, may be exacerbated in the context of COVID-19, as decisions about human resources, deployment and response activities may be made quickly, under stress and without inclusive input from women and underrepresented populations. Female humanitarians continue to face gendered pay gaps, that may be greater for national compared to international staff [12], and their occupational risks may be further compounded by the gendered impacts of COVID-19. For example, they may be facing increased risk of intimate partner violence at home, and issues related to increased care-taking responsibilities; both have been impacted by the COVID-19 outbreak and response measures [13]. Mental health problems and stress are common adverse outcomes among workers responding to epidemics [14, 15].

Given that the frontline humanitarian workforce is primarily composed of women, their voices and needs must be adequately represented and prioritized in organizational policies and in response planning. Ensuring gender equitable access to PPE and information is imperative, yet insufficient on its own. Humanitarian organizations need to apply gender-based analysis to examine existing policies and response plans to identify gaps in support for female workers. As an immediate measure, these organizations need to ensure access to safe housing, equitable and adequate pay, childcare and appropriate safety measures when at work and during deployment. Essential workers should be granted insurance, medical and family benefits, psychosocial support and resources to mitigate the risks of intimate partner violence. COVID-19 specific organizational procedures are needed to protect special populations within the workforce, including pregnant staff. At the operational level, partnering with communities to address mistrust, misinformation and stigma toward humanitarian workers will be critical to ensure protection from harassment and violence. Ultimately, addressing gender biases is necessary to foster professional standards and culture that elevates the position of female humanitarian staff to ensure their insights and specific needs are considered, and that promotes women’s leadership and equitable participation in decision-making.

Maintaining essential services will be critical to reduce COVID-19 deaths as well as adverse health effects among displaced populations. This will require understanding and prioritizing the health needs of displaced populations, as well as ensuring the safety of humanitarian workers and access to affected populations.

## Data Availability

Not applicable.
